# Health and sociodemographic determinants of excess mortality in Spanish nursing homes during the COVID-19 pandemic: a 2-year prospective longitudinal study

**DOI:** 10.1007/s00391-024-02294-4

**Published:** 2024-04-16

**Authors:** Anna Escribà-Salvans, Javier Jerez-Roig, Pau Farrés-Godayol, Dyego Leandro Bezerra de Souza, Dawn A. Skelton, Eduard Minobes-Molina

**Affiliations:** 1 Research group on Methodology, Methods, Models and Outcomes of Health and Social Sciences (M3O). Faculty of Health Sciences and Welfare. Centre for Health and Social Care Research (CESS), University of Vic-Central University of Catalonia (UVic-UCC), C. Sagrada Família 7, 08500 Vic, Spain; 2 Institute for Research and Innovation in Life Sciences and Health in Central Catalonia (IRIS-CC), Vic, Spain; 3 Department of Health Promotion and Rehabilitation, Institute of Sport Science and Innovations, Lithuanian Sports University, Kaunas, Lithuania; 4 Department of Collective Health, Federal University of Rio Grande do Norte, Natal, Rio Grande do Norte Brazil; 5 Research Centre for Health (ReaCH), School of Health and Life Sciences, Glasgow Caledonian University, Glasgow, UK

**Keywords:** Predictive factors, Survival, Aged, Long-term care, Pandemic, Vorhersagefaktoren, Überleben, Alter, Langzeitpflege, Pandemie

## Abstract

**Background:**

Age, multimorbidity, immunodeficiency and frailty of older people living in nursing homes make them vulnerable to COVID-19 and overall mortality.

**Objective:**

To estimate overall and COVID-19 mortality parameters and analyse their predictive factors in older people living in nursing homes over a 2-year period.

**Method:**

*Design*: A 2-year prospective longitudinal multicentre study was conducted between 2020 and 2022.

*Setting*: This study involved five nursing homes in Central Catalonia (Spain).

*Participants*: Residents aged 65 years or older who lived in the nursing homes on a permanent basis.

*Measurements*: Date and causes of deaths were recorded. In addition, sociodemographic and health data were collected. For the effect on mortality, survival curves were performed using the Kaplan-Meier method and multivariate analysis using Cox regression.

**Results:**

The total sample of 125 subjects had a mean age of 85.10 years (standard deviation = 7.3 years). There were 59 (47.2%) deaths at 24 months (95% confidence interval, CI, 38.6–55.9) and 25 (20.0%) were due to COVID-19, mostly in the first 3 months. In multivariate analysis, functional impairment (hazard ratio, HR 2.40; 95% CI 1.33–4.32) was a significant risk factor for mortality independent of age (HR 1.17; 95% CI 0.69–2.00) and risk of sarcopenia (HR 1.40; 95% CI 0.63–3.12).

**Conclusion:**

Almost half of this sample of nursing home residents died in the 2‑year period, and one fifth were attributed to COVID-19. Functional impairment was a risk factor for overall mortality and COVID-19 mortality, independent of age and risk of sarcopenia.

## Abstract (de)

**Hintergrund:**

Alter, Multimorbidität, Immunschwäche und Gebrechlichkeit älterer Menschen, die in Pflegeheimen leben, machen sie anfällig für COVID-19 und erhöhen die Gesamtmortalität.

**Zielsetzung:**

Schätzung der Gesamt- und COVID-19-Mortalität und Analyse ihrer prädiktiven Faktoren bei älteren Menschen, die in Pflegeheimen leben, über einen Zeitraum von 2 Jahren.

**Methode:**

Diese prospektive, multizentrische Längsschnittstudie wurde zwischen 2020 und 2022 in 5 Pflegeheimen in Zentral-Katalonien (Spanien) durchgeführt. Teilnehmer waren Bewohner im Alter von 65 Jahren oder älter, die dauerhaft in den Pflegeheimen lebten. Datum und Ursachen der Todesfälle wurden erfasst. Darüber hinaus wurden soziodemografische und gesundheitliche Daten erhoben. Für die Auswirkung der Sterblichkeit wurden Überlebenskurven nach der Kaplan-Meier-Methode und eine multivariate Analyse mittels Cox-Regression durchgeführt.

**Ergebnisse:**

Die Gesamtstichprobe von 125 Personen hatte ein Durchschnittsalter von 85,10 Jahren (Standardabweichung = 7,3). Nach 24 Monaten traten 59 (47,2 %) Todesfälle auf (95 % Konfidenzintervall [KI] 38,6–55,9), von denen 25 (20,0 %) auf COVID-19 zurückzuführen waren, zumeist in den ersten 3 Monaten. In der multivariaten Analyse war die Funktionseinschränkung (Hazard-Ratio [HR]: 2,40; 95 % KI 1,33–4,32) ein signifikanter Risikofaktor für die Sterblichkeit, unabhängig vom Alter (HR: 1,17; 95 % KI 0,69–2,00) und dem Risiko der Sarkopenie (HR: 1,40; 95 % KI 0,63–3,12).

**Schlussfolgerung:**

Fast die Hälfte dieser Stichprobe von Pflegeheimbewohnern starb innerhalb von 2 Jahren, ein Fünftel davon wurde auf COVID-19 zurückgeführt. Funktionseinschränkungen waren ein Risikofaktor für die Gesamt- und COVID-19-Mortalität, unabhängig von Alter und Sarkopenierisiko.

## Introduction

The coronavirus disease 2019 (COVID-19) pandemic began to have a major impact on society in 2019 [1] having unprecedented consequences on global health and economic systems.

In developed European countries with a very high older population the COVID-19 mortality was 83.7% for people > 70 years and 16.2% for people younger than 69 years in 2020 [2] with a higher prevalence of COVID-19 deaths in nursing homes (NH) [3]. Health problems and geriatric syndromes associated with ageing also determined the risk of mortality [4–9].

At the beginning of the pandemic Spain had a total of 326,613 people institutionalised in NHs [10] and a study conducted in Madrid reported a 14% mortality rate in older adults with COVID-19 in NHs [7]. The COVID-19 mortality has already been extensively studied in several countries, although most of these follow-ups have not exceeded 1 year [11]. Longer follow-ups would enable more accurate data and the identification of predictive factors that may be relevant to clinical practice.

The main aim of the study was to estimate overall and COVID-19 mortality parameters and analyse their predictive factors in older people living in NHs over a 2-year period.

## Methodology

### Study design and population

This is a 2-year multicentre observational cohort study. The study was conducted in five NH in Central Catalonia, Spain. It was designed following the Strengthening the Reporting of Observational studies in Epidemiology (STROBE) standards for cohort studies [12, 13]. Residents aged 65 years and older permanently living in NH were included. Those in a coma or palliative care (short-term prognosis), those who refused (or their legal guardian) to participate in the study and those who left the NH during the 2‑year cohort period were excluded.

### Sample size

For the calculation of the sample, the article by Burgaña Agoües et al. (2021) was taken as a reference due to its methodological similarity to the present article: Burgaña studied pandemic mortality due to COVID-19 in Spain, in people over 65 years of age resident in NH. Considering the findings of Burgaña Agoües et al. (2021) [14], i.e. the difference in proportions between individuals with severe functional impairment (23.0%) and deceased (11.1%), and with a confidence interval (CI) of 95% and a power of 80%, a sample size of 122 participants was estimated.

### Study procedures

The primary outcomes were all-cause mortality and COVID-19 over the 2‑year follow-up period. The mortality registry included cause and date of death, deaths in total, deaths due to COVID-19 including confirmed cases, and deaths due to symptomatic suspicion of COVID-19 [15]. Additional COVID-19 data collected included the presence of the disease, whether they had symptoms [16], the performance of COVID-19 screening tests such as C‑reactive protein (CRP), serological tests, and/or rapid antigen tests for SARS-CoV‑2 (RAT) [17]. The information was collected through on-line interviews with NH professionals as due to COVID-19, they could not be accessed.

Sociodemographic and health information was obtained from health centre records and cross-checked with health professionals. All sociodemographic variables and those described in this article were collected at baseline (January 2020), just before the onset of the COVID-19 pandemic in Spain. Falls (number) during the last year were obtained from NH registers. Nutritional status was assessed using the mini nutritional assessment (MNA) [18]. The SARC‑F [19] was used to identify individuals at risk of developing sarcopenia. Functional capacity was measured using the modified Barthel index and results were classified according to the degree of dependency as: independent, slightly dependent, moderately dependent, severely or totally dependent [20]. Continence status was reported using section H of the minimum data set (MDS) version 3.024 [21]. Cognitive status was assessed using the Pfeiffer scale [22] and frailty using the clinical frailty scale (CFS) [23]. Sedentary behaviour (SB) and waking-time movement behaviours (WTMB) were assessed using the ActivPAL 3TM activity monitor (PAL Technologies Ltd., Glasgow, UK) [24].

The study was conducted over a 2-year period and ended in March 2022.

### Statistical analysis

The nominal and ordinal quantitative variables were expressed according to frequency in percentages and the quantitative variables with mean and standard deviation (SD). Survival curves were formed using the Kaplan-Meier method and multivariate analysis was performed by Cox regression, using the hazard ratio (HR) as the measure of effect. The Statistical Package for the Social Sciences 27 (SPSS Inc., Chicago, IL, USA) was used for the analysis.

## Results

**Fig. 1:**
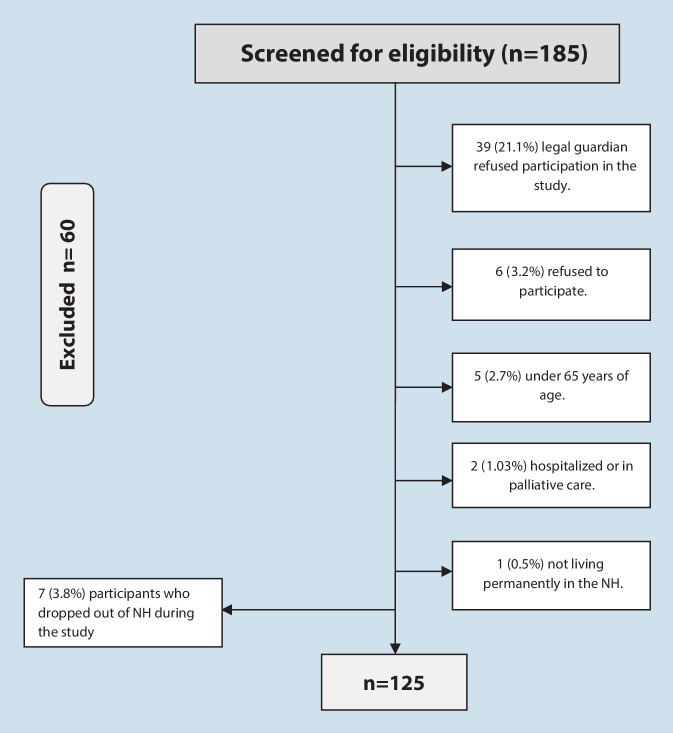
Flow chart of the sampling process

We recruited 125 people, 67.6% of the total number of NH residents in the main study. Finally, 7 (3.8%) participants who left NHs to reside elsewhere were excluded (Fig. 1).

The mean age of the participants was 85.1 years (SD = 7.3 years) and 104 (83.2%) were female. The mean number of months living in NH was 27.5 months (SD = 112.14 months). The analysis of health and sociodemographic variables is described in Table 1.

**Table 1:** Descriptive analysis of the sample of institutionalized older adults living in nursing homes of Central Catalonia, Spain (*n* = 125)

Variables	Frequency (%)/mean (SD)
*NH type*	
State subsidized places	40 (32.0)
Private	85 (68.0)
Smoking	6 (4.8)
Drinkers	9 (12.7)
Chronic conditions	5.0 (2.46 SD)
Hypertension	80 (64.0)
Dementia	68 (54.4)
Cardiac pathology	51 (40.8)
Depression	36 (28.8)
Diabetes mellitus tape 2	36 (28.8)
Renal pathology	32 (25.6)
CVA	25 (20.0)
Cancer	23 (18.4)
Pulmonary pathology	22 (17.6)
*Urinary incontinence*	
Yes	87 (69.6)
No	35 (28.0)
Unclassifiable	3 (2.4)
*Fecal incontinence*	
Yes	36 (28.8)
No	87 (69.6)
Unclassifiable	2 (1.6)
Fall/s in previous year	58 (46.4)
*Nutritional status*	
Involuntary weight loss	25 (13.5)
Risk of malnutrition or malnourished	56 (44.8)
Obesity	78 (62.4)
Risk of sarcopenia	94 (75.2)
*Functional impairment*	
Independent	7 (5.6)
Slightly dependent	47 (37.6)
Moderately dependent	19 (15.2)
Severely or totally dependent	52 (41.6)
*Cognition*	
No cognitive deficit	10 (8.0)
Mild cognitive deficit	20 (16.0)
Moderate cognitive deficit	24 (19.2)
Severe cognitive deficit	58 (46.4)
Unknown	13 (10.4)
*SB and WTMB*	
Waking hours	11.0 (1.5 SD)
% of waking time in SB	82.6% (17.5 SD)
Hours in upright position (standing and walking)	1.6 (1.7 SD)
Steps per day	1.345 (2417.4 SD)
Sit to stand transitions per day	18.2 (18.3 SD)
Hospitalization	26 (0.43 SD)

*SD* standard deviation, *NH* nursing homes, *CVA* cerebral vascular accident, *SB* sedentary behaviour, *WTMB* waking-time movement behaviour

In the 2‑year period from baseline to the end of the study, 59 participants (47.2%) died, of whom 25 (20.0%) died from COVID-19 and 34 (27.2%) from other causes. All COVID-19 deaths occurred in the first year of the study: 44 (74.5%) of the 59 individuals had already died within the first 90 days (at the peak of COVID-19).

### Survival and associated factors according to the variable mortality

In the bivariate analysis, mortality was associated with functional impairment, urinary incontinence (UI), faecal continence, risk of sarcopenia, % of waking time in SB and with a *p*-value of less than 0.05. All other health and sociodemographic variables were not significant (Table 2).

**Table 2:** Association of variables to health, functional and sociodemographic^1^ with mortality (*n* = 125)

Variables	*n* (%)	Number of deaths	Number of survivors	Probability of death (%)	*p (Log rank)*
*Functional impairment* (*n* = 125)					
No/mild or moderate	73 (58.4)	24	49	65.3	0.001*
Total impairment	52 (41.6)	35	17	31.6	
*Urinary incontinence* (*n* = 122)					
No	35 (28.0)	10	25	66.8	0.008*
Yes	87 (69.6)	49	38	43.2	
*Risk of sarcopenia *(*n* = 125)					
No	31 (24.8)	9	22	71.0	0.018*
Yes	94 (75.2)	50	44	45.0	
*% waking time in SB *(*n* = 84)					
≤ 85%	41 (48.8)	11	30	73.2	0.028*
> 85%	43 (51.2)	22	21	48.4	
*Fecal incontinence *(*n* = 123)					
No	87 (70.7)	35	52	57.9	0.029*
Yes	36 (29.3)	22	14	38.4	
*NH type* (*n* = 125)					
State subsidized places	40 (32.0)	14	26	64.8	0.076
Private	85 (68.0)	45	40	46.1	
*Nutritional status *(*n* = 79)					
Normal	23 (29.2)	7	16	69.6	0.082
At risk or malnourished	56 (70.8)	31	25	43.6	
*Drinkers* (*n* = 71)					
No	62 (87.3)	34	32	45.5	0.249
Yes	9 (12.7)	1	4	80.0	

*SB* sedentary behaviour, *NH* nursing homes

^1^ With a *p* value lower than 0.250

* Statistically significant (< 0.05)

The variables were tested for collinearity and none of them showed collinearity with each other. A multivariate analysis was performed with the model with adjusted values including the variables age with severe functional impairment and risk of sarcopenia. The result showed that functional impairment predicted mortality independently of age (which was not statistically significant) and sarcopenia risk (Table 3).

**Table 3:** Survival univariate and multivariate Cox analysis^1^ in older people living in NHs (*n* = 125)

Variables	HR (ref.)	CI (95%)	*p (Cox)*	HR (ref.)	CI (95%)	*p (Cox)*
	*Univariate analysis*			*Multivariate analysis*		
*Functional impairment *(*n* = 125)						
No/mild or moderate	–	–	–	–	–	–
Total impairment	2.77	(1.64–4.67)	0.001*	2.40	(1.33–4.32)	0.003*
*Urinary incontinence* (*n* = 122)						
Yes	–	–	–	–	–	–
No	2.43	(1.23–4.81)	0.010*	–	–	–
*Risk of sarcopenia* (*n* = 125)						
Yes	–	–	–	–	–	–
No	2.29	(1.12–4.66)	0.022*	1.40	(0.63–3.12)	0.403
*Fecal incontinence* (*n* = 123)						
No	–	–	–	–	–	–
Yes	1.80	(1.05–3.07)	0.031*	–	–	–
% *waking time in SB* (*n* = 84)						
≤ 85%	–	–	–	–	–	–
> 85%	2.20	(1.07–4.55)	0.033*	–	–	–
*NH type* (*n* = 125)						
Private	–	–	–	–	–	–
State subsidized places	0.85	(0.32–1.06)	0.080	–	–	–
*Nutritional status* (*n* = 79)						
Normal	–	–	–	–	–	–
Risk or malnourished	2.04	(0.89–4.64)	0.089	–	–	–
*Age* (years, *n* = 125)						
≤ 85	–	–	–	–	–	–
> 86	1.33	(0.78–2.26)	0.284	1.17	(0.69–2.00)	0.549

*HR* hazard ratio, *CI* confidence interval, *SB* sedentary behaviour, *NH* nursing homes

^1^ Variables with a *p* value lower than 0.250 in univariate analysis are shown

* Statistically significant (*p* < 0.05)

### Survival and associated factors according to the variable COVID-19 or other-cause mortality

**Fig. 2:**
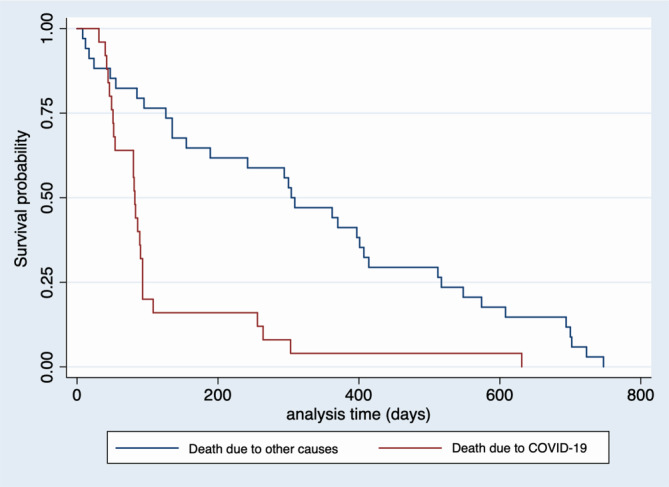
Kaplan-Meier survival estimates

In the univariate analysis, functional impairment, living in a private NH, being older than 86 years, malnutrition and being female were risk factors for COVID-19 mortality. Functional impairment was associated with mortality from other health causes with a *p*-value of less than 0.050 (Tables 4 and 5; Fig. 2).

**Table 4:** Analysis of the association of health and sociodemographic variables^1^ NH residents with mortality (COVID-19 or other causes) (*n* = 59)

Variables	*n *(%)	Deaths for covid-19 (number of events)	Deaths due to other causes (number of events)	Probability of death (%)	*p (Log rank)*
*Nutritional status* (*n* = 38)					
Normal	7 (18.4)	5	2	28.6	0.022*
Risk or malnourished	31 (81.6)	10	21	61.8	
*Sex* (*n* = 59)					
Male	9 (15.2)	6	3	14.6	0.041*
Female	50 (84.7)	19	31	29.8	
*Functional impairment* (*n* = 59)					
No/mild or moderate	24 (40.6)	15	9	26.1	0.045*
Total impairment	35 (59.3)	10	25	63.5	
% *waking time in SB* (*n* = 33)					
≤ 85%	11 (33.3)	7	4	36.4	0.082
> 85%	22 (66.6)	6	16	68.7	
*Fecal incontinence* (*n* = 57)					
No	35 (61.4)	18	17	37.6	0.132
Yes	22 (38.6)	6	16	68.2	
*Hospitalizations* (*n* = 46)					
No	34 (73.9)	17	17	32.0	0.194
Yes	12 (26.1)	4	8	58.2	

*SB* sedentary behaviour, *NH* nursing homes

^1^ With a *p* value lower than 0.250

* Statistically significant (< 0.05)

**Table 5:** Univariate Cox analysis: association of COVID-19 mortality with other causes of death in relation to covariables^1^ in older people living in NHs (*n* = 59)

Variables	HR ref. (exp^B^)	CI (95%)	*p* value
	*(Univariate analysis)*		
*Functional impairment* (*n* = 59)			
No/mild or moderate impairment	–	–	**–**
Total impairment	2.04	(1.40–2.97)	0.001*
*NH* type (*n* = 59)			
State subsidized places	–	–	–
Private	1.73	(1.13–2.66)	0.012*
*Age* (years, *n* = 59)			
≤ 85	–	–	–
≥ 86	1.47	(1.03–2.11)	0.035*
*Fecal incontinence* (*n* = 57)			
No	–	–	–
Yes	1.47	(0.99–2.18)	0.055
*Urinary incontinence* (*n* = 59)			
Yes	–	–	–
No	1.41	(0.95–2.08)	0.090
% *waking time in SB* (*n* = 33)			
≤ 85%	–	–	–
> 85%	1.36	(0.88–2.09)	0.161

*HR* hazard ratio, *CI* confidence interval, *NH* nursing homes, *SB* sedentary behaviour

^1^ Variables with a *p* value lower than 0.250 are shown

* Statistically significant (< 0.05)

We tested for collinearity between the variables and none of them were collinear with each other. The number of individuals in this variable is 59. Different combinations of significant variables, such as functionality, age and type of NH among others, were tested by multivariate analysis, but no significant results were found (Table 5).

## Discussion

The main objective of this study was to examine the incidence of all-cause and COVID-19 mortality and to analyse the predictive factors in older NH residents over a 2-year period since the onset of the pandemic.

The results indicate that almost half of participants died with 20% being attributed to COVID-19. Most of the deaths (74.5%) were in the first 3 months of the study, coinciding with the outbreak of the COVID-19 pandemic in Spain. In the second year of the study, the survival curve became horizontal again, after the implementation of preventive measures. This study shows a higher incidence of mortality in the 1‑year period than other studies. Several articles on the pandemic phase report data on excess mortality [11]. A study in Barcelona reported a 3-month COVID-19 mortality rate of 11.1% in institutionalised older people [14]. For deaths from other causes, they reported excess mortality among institutionalised cases (34.8%) [14].

We also report the association of health, social and demographic variables with mortality: functional impairment, UI, sarcopenia risk and % of waking time in SB were found to be factors associated with mortality and functional impairment, type of NH and age were associated with COVID-19 mortality. The literature shows that UI and risk of sarcopenia are associated with mortality [25–27] and those who spent more time in SB had a higher risk of mortality [28].

Unlike other studies, our data show an increase of mortality in private NHs [29]. The NH type and size influenced mortality during the COVID-19 pandemic in other studies [29]. Braun et al. (2020) attributed these results to the lack of organisation and shortage of personal protective equipment (PPE) in private NHs [29].

This study has the limitation of the COVID-19 pandemic, which impeded access to NHs, increased deaths in older people and did not enable us to have a larger sample.

The strength of the study lies in the telematic data collection in NHs. This enabled us to extract real information on the health and social status of residents. The fact that we collected data prior to the start of the pandemic allowed us to make a comparison of the health and sociodemographic status of institutionalised older people. By having a cross-sectional analysis of the prepandemic sample, we provide data on the factors that predicted the risk of dying in a 2-year follow-up.

## Conclusion

Almost half of this sample of NH residents died during the 2‑year observation period. One fifth of deaths were attributed to COVID-19 mostly in the first quarter, coinciding with the peak of the pandemic. Functional impairment was a risk factor for overall mortality and COVID-19 mortality, independent of age and risk of sarcopenia.

## Data Availability

The datasets used and/or analyzed during the current study are available from the corresponding author on reasonable request.

## Abbreviations

AHT: Arterial hypertension
BMI: Body mass index
CFS: Clinical frailty scale
CI: Confidence interval
COVID-19: Coronavirus disease
CVA: Cerebral vascular accident
HR: Hazard ratio
ICC: Interclass correlation coefficient
MDS: Minimum data set
MNA: Mini nutritional assessment
NH: Nursing homes
PCR: C‑reactive protein
PPE: Personal protective equipment
RAT: Rapid antigen tests
SARC‑F: Questionnaire assistance in walking, rising from chair, climbing stairs and falling
SB: Sedentary behaviour
SD: Standard deviation
SPSS: Statistical Package Social Sciences
STROBE: Strengthening the Reporting of Observational Studies in Epidemiology
UI: Urinary incontinence
WTMB: Waking-time movement behaviours

## Declarations

Ethical approval was obtained from the Ethics and Research Committee of the University of Vic—Central University of Catalonia (registration number 92/2019 and 109/2020). Signed informed consent was obtained from the residents or the legal guardians. The project meets the criteria required in the Helsinki Declaration as well as the Organic Law 3/2018 (5 December) on the Protection of Personal Data and Guarantee of Digital Rights.
